# Fostering an Understanding of Interprofessional Approaches to Geriatrics

**DOI:** 10.1093/geroni/igaa057.1436

**Published:** 2020-12-16

**Authors:** Anna Faul, Pamela Yankeelov, Sam Cotton

**Affiliations:** University of Louisville, Louisville, Kentucky, United States

## Abstract

Serving older adults with multiple chronic conditions and variable social, emotional, or physical support effectively within the primary care setting requires an interdisciplinary approach to care, together with the integration of novel approaches to care coordination (Dorr et al, 2006). The purpose of this study is to examine the use of interprofessional learning models to educate a healthcare workforce that meets the needs of older adults by integrating geriatrics with primary care, maximizing patient engagement, and transforming the healthcare system. Specifically, the targeted learners for this curriculum were from a healthcare system in Belize that had no previous specialty training in interprofessional geriatrics care. The 4-day training took place in Belize with an interprofessional group of healthcare professionals that included social work, nursing and medicine. 100 learners participated in the trainings and including participants from social work, nursing and medicine. To evaluate the program, Kirkpatrick’s Training Evaluation Model (Kirkpatrick & Kirkpatrick, 2005) was used to determine if learners were satisfied with the content (reaction), skilled (knowledge & skill) and confident in their abilities to utilize the curriculum (application of knowledge & skills). Analysis showed that learners, irrespective of discipline, were satisfied with the program. All disciplines experienced significant differences in their self-efficacy with working on interdisciplinary teams from pre to post assessments. Specifically, there was an increase in learner’s confidence related to learning to work together cooperatively with other professions and how to communicate effectively with other members of an interprofessional team. Implications for future interprofessional curriculum will be discussed.

